# The Effects of Antipsychotic Treatment on Presynaptic Dopamine Synthesis Capacity in First-Episode Psychosis: A Positron Emission Tomography Study

**DOI:** 10.1016/j.biopsych.2018.07.003

**Published:** 2018-07-11

**Authors:** Sameer Jauhar, Mattia Veronese, Matthew M. Nour, Maria Rogdaki, Pamela Hathway, Sridhar Natesan, Federico Turkheimer, James Stone, Alice Egerton, Philip McGuire, Shitij Kapur, Oliver D. Howes

**Affiliations:** a Department of Psychological Medicine, Institute of Psychiatry, Psychology and Neuroscience, King’s College London, London, United Kingdom; b Centre for Neuroimaging Sciences, Institute of Psychiatry, Psychology and Neuroscience, King’s College London, London, United Kingdom; c Psychosis Studies, Institute of Psychiatry, Psychology and Neuroscience, King’s College London, London, United Kingdom; d Early Intervention Psychosis Clinical Academic Group, South London and Maudsley NHS Trust, London, United Kingdom; e Psychiatric Imaging Group, MRC London Institute of Medical Sciences, Imperial College, Hammersmith Hospital, London, United Kingdom; f Institute of Clinical Sciences, Faculty of Medicine, Imperial College, Hammersmith Hospital, London, United Kingdom; g Faculty of Medicine, Dentistry and Health Sciences, University of Melbourne, Parkville, Victoria, Australia

**Keywords:** Antipsychotic drugs, Dopamine, F-DOPA, Positron emission tomography, Psychosis, Schizophrenia

## Abstract

**Background:**

Elevated striatal dopamine synthesis capacity has been implicated in the etiology and antipsychotic response in psychotic illness. The effects of antipsychotic medication on dopamine synthesis capacity are poorly understood, and no prospective studies have examined this question in a solely first-episode psychosis sample. Furthermore, it is unknown whether antipsychotic efficacy is linked to reductions in dopamine synthesis capacity. We conducted a prospective [^18^F]-dihydroxyphenyl-L*-*alanine positron emission tomography study in antipsychotic naïve/free people with first-episode psychosis commencing antipsychotic treatment.

**Methods:**

Dopamine synthesis capacity (indexed as influx rate constant) and clinical symptoms (measured using Positive and Negative Syndrome Scale) were measured before and after at least 5 weeks of antipsychotic treatment in people with first-episode psychosis. Data from a prior study indicated that a sample size of 13 would have >80% power to detect a statistically significant change in dopamine synthesis capacity at alpha = .05 (two tailed).

**Results:**

A total of 20 people took part in the study, 17 of whom were concordant with antipsychotic medication at therapeutic doses. There was no significant effect of treatment on dopamine synthesis capacity in the whole striatum (*p* = .47), thalamus, or midbrain, nor was there any significant relationship between change in dopamine synthesis capacity and change in positive (ρ = .35, *p* = .13), negative, or total psychotic symptoms.

**Conclusions:**

Dopamine synthesis capacity is unaltered by antipsychotic treatment, and therapeutic effects are not mediated by changes in this aspect of dopaminergic function.

SEE COMMENTARY ON PAGE e1

Psychotic disorders such as schizophrenia have a lifetime prevalence of more than 1% and are a major cause of disease burden in young adults (1). Antipsychotic medications (dopamine D_2/3_ receptor antagonists) are the mainstay of treatment for acute psychosis and reduce risk of relapse in the longer term (2). Antipsychotics, however, have limited efficacy in a substantial proportion of patients from illness onset 3, 4, 5. The use of antipsychotics is also limited by poor tolerability (6). Thus, there is a need to understand the neurobiology underlying treatment response to guide development of alternative treatments (7).

The dopamine hypothesis is one of the leading neurobiological hypotheses of psychosis 8, 9, 10, 11. A meta-analysis of imaging studies found that the predominant dopamine abnormalities seen in schizophrenia involve the presynaptic dopamine system, including elevated striatal dopamine synthesis capacity (indexed as influx rate constant [Ki^cer^]), using [^18^F]-dihydroxyphenyl-L*-*alanine ([^18^F]-DOPA) positron emission tomography (PET) (12). Moreover, striatal dopamine synthesis capacity increases longitudinally with onset of psychosis (13), and striatal dopamine release is greater in patients who are acutely unwell relative to stable patients (14). Added to cross-sectional studies in which dopamine synthesis capacity has been related to psychotic symptoms 15, 16, this suggests dopamine function to have a state component in psychosis.

Consistent with these findings and the dopamine hypothesis, all antipsychotic drugs are dopamine D_2/3_ receptor blockers 17, 18, 19. Antipsychotic drugs may also act presynaptically to reduce dopamine neuron firing, and this could be their primary mode of therapeutic action (20). Support for this comes from rodent studies, which have shown that subchronic treatment with haloperidol and a number of other first-generation antipsychotics, as well as second-generation antipsychotics, induces depolarization blockade of dopamine neurons 20, 21. Prolonged treatment leads to decreased firing of dopamine neurons in substantia nigra and ventral tegmental area (22). The effects of antipsychotics on dopamine neuron firing have also been tested in a neurodevelopmental animal model of schizophrenia (the methylazoxymethanol acetate model) that shows increased population activity of midbrain dopamine neurons. This study showed that depolarization blockade is induced more rapidly in these animals than in wild-type animals (23), suggesting that depolarization blockade may be more rapid in a hyperdopaminergic state. This contrasts with findings from an animal study using microdialysis, which showed no effect of chronic haloperidol treatment on striatal levels of extracellular dopamine and its metabolites (24).

To date, only one study has examined effects of subchronic antipsychotic treatment in people with psychosis. Gründer *et al.* found decreased [^18^F]-DOPA uptake (k3) in striatum and thalamus in 9 antipsychotic-free people with schizophrenia treated with haloperidol (25). That study also found that greater improvement in negative symptoms, but not positive symptoms, was associated with greater reduction in thalamic [^18^F]-DOPA uptake. This suggests that reduction in dopamine synthesis capacity may be related to symptom change. However, the study by Gründer *et al.* used haloperidol, a first-generation antipsychotic, at relatively high doses (mean dose of 8.9 mg/day)—a dose that would be expected to result in D_2/3_ occupancy >90% in first-episode patients (17), which might explain effects on the dopamine system. Modern clinical practice, however, involves treatment with second-generation antipsychotics at lower relative doses, which would be expected to have lower D_2/3_ occupancy (26). It therefore remains unknown whether antipsychotic treatment with second-generation antipsychotics at doses reflecting current practice decreases striatal dopamine synthesis capacity and whether this has any relationship to symptom change.

Therefore, we sought to examine the effect of antipsychotic medication on striatal dopamine synthesis capacity in people experiencing their first episode of psychotic illness and its relationship to symptom change.

We tested the following hypotheses: 1) There would be a decrease in striatal dopamine synthesis capacity with antipsychotic treatment; and 2) Reduction in striatal dopamine synthesis capacity would be directly associated with reduction in positive psychotic symptoms, and reduction in thalamic dopamine synthesis capacity would be directly associated with negative psychotic symptom change.

An exploratory analysis of associative striatum baseline Ki^cer^ and symptomatic response was also conducted based on a prior cross-sectional study suggesting a relationship between this striatal subdivision and antipsychotic response (27).

## Methods and Materials

Ethical permission was obtained from the East of England–Cambridge East Ethics Committee and the Administration of Radioactive Substances Advisory Committee. All participants provided informed written consent to participate.

### Participants

Patients were recruited from first-episode psychosis services in London. Inclusion criteria were diagnosis of a psychotic disorder according to ICD-10 criteria (28), fulfilling criteria for having a first episode of psychosis (29), requiring treatment with antipsychotic medication, and being antipsychotic naïve or antipsychotic free for at least 6 weeks [other clinical studies in similar populations require being antipsychotic free for a minimum of 3 weeks 30, 31].

For comparison, a matched sample of healthy control subjects was included. Inclusion criteria included no psychiatric morbidity, as assessed by the Mini-International Neuropsychiatric Interview (32), and no contraindications to PET scanning, as per the patient sample.

Exclusion criteria for all subjects were history of significant head trauma, dependence on illicit substances or alcohol, medical comorbidity (other than minor illnesses), use of sodium valproate [owing to effects on dopamine synthesis capacity (33)], and contraindications to scanning (such as pregnancy).

Tobacco smoking was not an exclusion criterion.

### Clinical Measures

The following clinical measures were rated at baseline and at least 5 weeks after antipsychotic initiation: Positive and Negative Syndrome Scale (PANSS) (34), Global Assessment of Functioning (35), and Clinical Global Impression–Improvement scale (CGI-I) (36). Response status (dichotomized as responder vs. nonresponder) was measured, in keeping with prior PET studies, based on a rating of much improved or very much improved on CGI-I (17).

### Baseline

People presenting with first-episode psychosis received one baseline [^18^F]-DOPA scan prior to initiation of antipsychotic medication. They were classified as antipsychotic naïve or medication free (free of oral antipsychotic medication for 6 weeks or longer).

### Treatment

Because this was a naturalistic study, antipsychotic treatment was decided by the treating clinician and patient. All doses were required to be within the therapeutic range for the drug defined in the Maudsley Prescribing Guidelines (36). Use of other psychotropic medication (such as antidepressants and benzodiazepines) was permitted, although use of sodium valproate was not [because it may have effects on [^18^F]-DOPA uptake (33)]. To assess concordance with antipsychotic medication, we used a multisource approach, requiring evidence of adequate adherence on at least two of the following: antipsychotic plasma levels, pharmacy and electronic medical dispensing records, and report from the patient and an independent source (family member/caregiver or health care professional). Adequate concordance was defined as taking a minimum of 80% of prescribed doses, in line with consensus recommendations (37). To measure antipsychotic exposure, we determined chlorpromazine-equivalent dose years, calculated as described by Andreasen *et al.* (38). [In the cases of lurasidone and amisulpride, we used the method described by Leucht *et al.* (39), using data from the Maudsley Prescribing Guidelines, because these are not covered by Andreasen *et al.*].

### Follow-up

All participants received follow-up [^18^F]-DOPA scans and clinical measures (PANSS, Global Assessment of Functioning, and CGI-I) after at least 5 weeks of antipsychotic treatment at an adequate dose as defined in the Maudsley Prescribing Guidelines and meeting concordance criteria described above.

Clinical follow-up was conducted 6 months after the baseline scan to confirm diagnosis using the Mini-International Neuropsychiatric Interview.

### [^18^F]-DOPA PET Imaging

All participants were asked not to eat or drink (except water) and to refrain from alcohol for 12 hours prior to scan. Cigarette smokers were not permitted to smoke during the 4 hours preceding the scan. The 4-hour cutoff for last cigarette smoked in this study was based on evidence that nicotine’s occupancy of the acetylcholine nicotinic receptor does not change appreciably between 2 and 5 hours after last administration of nicotine (40).

Imaging data were obtained on a Siemens Biograph 6 HiRez PET scanner (Siemens, Erlangen, Germany) in three-dimensional mode. One hour before scanning, participants received 400 mg of entacapone, a peripheral catechol-*o*-methyl-transferase inhibitor, and 150 mg of carbidopa, a peripheral aromatic acid decarboxylase inhibitor, to prevent formation of radiolabeled metabolites that may cross the blood-brain barrier (41). Participants were positioned in the scanner with the orbitomeatal line parallel to the transaxial plane of the tomograph. Head position was marked and monitored, and movement was minimized using a head strap. After acquiring a computed tomography scan for attenuation correction, [^18^F]-DOPA was administered by bolus intravenous injection 30 seconds after start of PET imaging. PET data were acquired in 32 frames of increasing duration over the 95-minute scan (frame intervals: 8 × 15 seconds, 3 × 60 seconds, 5 × 120 seconds, 16 × 300 seconds).

The region-of-interest analysis was conducted blind to medication status. Our primary end point was striatal influx constant (Ki^cer^) for the whole striatum. For each participant, we calculated Ki^cer^ for bilateral whole striatum, caudate, putamen, and thalamus in light of prior work by Gründer *et al.* (25). Whole striatum was chosen instead of striatal functional subdivisions (such as associative striatum) given lack of prior investigation of striatal subdivisions and antipsychotic effects. Secondary analyses were also conducted with substantia nigra, given prior findings of a relationship between psychotic symptoms and dopamine synthesis capacity in substantia nigra (15), and with associative striatum and treatment response, based on prior literature (27).

Correction for head movement during scan was performed by employing a mutual information algorithm (42). SPM8 (43) was used to automatically normalize a tracer-specific [^18^F]-DOPA template (44) together with the striatal brain atlas as defined by Martinez *et al.* (45) and the Hammersmith brain atlas (46). The Hammersmith brain atlas was used to identify extrastriatal regions and reference region (cerebellum). The region-of-interest atlas was transformed into the subject’s PET space using the tracer-specific template without using coregistered magnetic resonance imaging. This method showed good reliability in a previous test–retest study (47).

Ki^cer^ was calculated using the Patlak–Gjedde graphical approach adapted for a reference tissue input function (48). Further details of the image analysis approach are given in prior publications 16, 49. Although our reference region approach is robust to global differences in radiotracer delivery to the brain 50, 51, we examined the reference region (cerebellum) to ascertain change in standardized uptake value in cerebellum at 95 minutes. To exclude potential effects of weight, we conducted correlation analysis of weight and Ki^cer^ for participants at baseline.

Striatal volume measures were derived from the atlas-based segmentation as the number of voxels in the striatal region multiplied by the volume of a single PET image voxel (voxel volume = 2.05 mm × 2.05 mm × 2 mm = 8.41 mm^3^). This analysis was undertaken to investigate whether there was a change in striatal volume over time.

### Statistical Analysis

Statistical analyses were performed using SPSS Version 23 (IBM Corp., Armonk, NY), and significance was set at *p* < .05 (two tailed). Normality of distribution for dopamine synthesis capacity (Ki^cer^), PANSS ratings, and changes in all these measures was assessed using the Shapiro-Wilk test. To test hypothesis 1 (change in Ki^cer^ with antipsychotic medication) and changes in clinical variables over follow-up, we used paired sample *t* tests. The data from Gründer *et al.* (25) indicated that a sample size of 13 would have >80% power to detect a statistically significant change in dopamine synthesis capacity at alpha = .05 (two tailed).

To test hypothesis 2 (relationship between change in Ki^cer^ and change in PANSS symptoms scores), we used Pearson’s correlation coefficients for normally distributed data and Spearman’s correlation coefficients for non-normally distributed data. Because hypothesis 2 related to the relationship between dopamine synthesis capacity and symptom change irrespective of treatment (examining possible state effects), all subjects who took part in the study were included (including those nonconcordant with antipsychotic medication and those receiving inadequate antipsychotic treatment) in the primary analysis. We then conducted a further exploratory analysis to determine whether correlations between dopamine synthesis capacity and symptom change were seen in patients who met full adherence criteria to test specificity to antipsychotic treatment.

Change in clinical symptom scales was measured by calculating percentage change in PANSS score, accounting for minimum scores (7 for positive and negative symptoms and 30 for total symptoms) as shown here for the PANSS positive symptom subscale:

$$\text{\%}\;\text{change}\;\text{in}\;\text{positive}\;\text{PANSS}=\;\frac{\left(\left(\text{baseline}\;\text{score}-7\right)\;-\;\left(\text{follow}-\text{up}\;\text{score}\;-\;7\right)\right)\;\text{∗}\;100}{\left(\text{baseline}\;\text{score}\;-\;7\right)}$$

Change in dopamine synthesis capacity (DSC) was calculated as follows:

$$\text{Change}\;\text{in}\;\text{DSC}=\;\frac{{\text{DSC}}_{\text{baseline}}-{\;\text{DSC}}_{\text{follow}-\text{up}}}{{\text{DSC}}_{\text{baseline}}}\;\text{∗}100$$

## Results

A total of 20 patients completed the study and received two [^18^F]-DOPA scans. Of these, 15 were antipsychotic naïve and 5 were medication free at time of scanning (previously receiving antipsychotic medication and being medication free for 6 weeks or longer). Of these patients, 3 did not meet the criteria for adequate treatment prior to the follow-up scan (1 patient was treated with a subtherapeutic dose of antipsychotic medication because that patient declined to take higher doses, and 2 patients were nonconcordant).

Therefore, we excluded these 3 patients, leaving 17 patients to test hypothesis 1 (change in Ki^cer^ after antipsychotic treatment).

All 20 patients were included to test hypothesis 2 (relationship between change in Ki^cer^ and change in symptoms) because this was not dependent on antipsychotic treatment. Demographic and clinical details are given in Table 1. Details of antipsychotic treatment that patients received after baseline scan are given in Supplemental Table S1.

**Table 1:** Demographic and Medication Status for Both PET Samples

Variable	PET Sample Adherent to Treatment (*n* = 17)	PET Sample Including Nonadherent or Partially Adherent Patients (*n* = 20)	Control Sample (*n* = 20)
Age, Years, Mean (SD)	24.00 (2.87)	24.55 (3.36)	24.55 (3.99)
Male Subjects (%)	*n* = 15 (88)	*n* = 17 (85)	*n* = 18 (90)
Ethnicity of Subjects (%)	White, *n* = 6 (35) Black, *n* = 5 (29) Asian, *n* = 2 (12) Mixed, *n* = 4 (24)	White, *n* = 8 (40) Black, *n* = 6 (30) Asian, *n* = 2 (10) Mixed, *n* = 4 (20)	White, *n* = 10 (50) Black, *n* = 6 (30) Asian, *n* = 1 (5) Mixed, *n* = 3 (15)
Smoking Status (%)	Current smoker, *n* = 9 (53) Past smoker, *n* = 3 (18) Nonsmoker, *n* = 5 (29)	Current smoker, *n* = 10 (50) Past smoker, *n* = 3 (15) Nonsmoker, *n* = 7 (35)	Current smoker, *n* = 8 (40) Past smoker, *n* = 3 (15) Nonsmoker, *n* = 9 (45)
Medication Status at Baseline	Antipsychotic naïve, *n* = 12 Antipsychotic free, *n* = 5	Antipsychotic naïve, *n* = 15 Antipsychotic free, *n* = 5	N/A

N/A, not applicable; PET, positron emission tomography.

Median time between scans was 71 days (interquartile range of 125 days).

No relationship was found between time between PET scans and change in Ki^cer^ (ρ = −.03, *p* = .92).

Of the 20 patients recruited in total, 12 met criteria for schizophrenia and 8 for bipolar affective disorder at 6-month follow-up.

There was a statistically significant elevation in whole striatal Ki^cer^ in patients compared with control subjects (*t*_38_ = 2.32, *p* = .03, two tailed) (control subjects mean = 12.41 × 10^−3^/min, SD = 1.1 × 10^−3^/min).

Data on the baseline scans for 18 patients reported here have been published in Jauhar *et al.* (16) and Jauhar *et al.* (52). None of the follow-up scans have been previously reported.

The latter study analyzed baseline Ki^cer^ in a larger group of first-episode psychosis patients (*n* = 26). The 8 additional subjects did not receive follow-up F-DOPA PET scans.

### Antipsychotic Treatment

Two patients started on an antipsychotic but discontinued it owing to side effects and switched to another antipsychotic that they then received at an adequate dose and duration prior to follow-up scan. Where this occurred, the discontinued medication is noted first, followed by the drug they then received. Three patients took low-dose aripiprazole to prevent weight gain and hyperprolactinemia in addition to the main antipsychotic. Where this occurred, the main antipsychotic is noted first, followed by aripiprazole to indicate its use as an adjunct. Regarding adjunctive medication, 1 patient was taking sertraline (150 mg) at initial scan, which was unchanged at follow-up.

There was a significant reduction in all symptoms following treatment (Table 2).

**Table 2:** Clinical and Imaging Details

Variable	PET Baseline, *n* = 17	PET Follow-up, *n* = 17	Difference Between Time Points^a^
Injected Activity, MBq	146.96 (4.33)	145.32 (4.41)	*Z* = −0.024, *p* = .98
Specific Activity, GBq/mmol	0.031 (0.005)	0.027 (0.011)	*t*_16_ = 1.47, *p* = .16
PANSS Positive	20.59 (6.98)	12.76 (5.02)	*Z* = −3.27, *p* < .05
PANSS Negative	17.24 (5.22)	12.47 (5.34)	*Z* = −3.00, *p* < .05
PANSS Total	77 (21)	47 (19.5)	*Z* = −3.52, *p* < .05

Values are presented as median (interquartile range).

GBq, gigabecquerel; MBq, megabecquerel; PANSS, Positive and Negative Syndrome Scale.

a Paired *t* test used for normally distributed data; Wilcoxon signed ranks test used for non-normally distributed data.

### Baseline Ki^cer^ and Change in PANSS

There was a significant positive relationship between baseline associative striatum Ki^cer^ and change in positive symptoms (*r* = .52, *p* = .03).

There was a significant positive relationship between baseline associative striatum Ki^cer^ and change in total symptoms (*r* = .49, *p* = .045).

There was a trend for an association between whole striatum Ki^cer^ and change in positive symptoms (*r* = .48, *p* = .05).

There was no association between whole striatum Ki^cer^ and change in total symptoms (*r* = .46, *p* = .06).

There was no association between whole striatum Ki^cer^ and change in negative symptoms (Spearman’s ρ = .37, *p* = .14).

### Change in Dopamine Synthesis Capacity With Antipsychotic Medication

**Figure 1:**
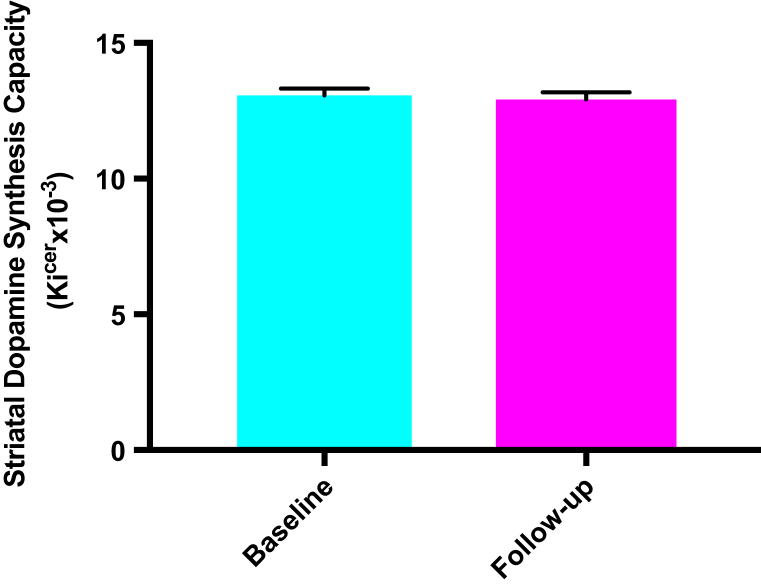
Dopamine synthesis capacity at baseline and follow-up showing group means and standard error of the mean. There was no significant change in dopamine synthesis capacity over time with antipsychotic treatment (*p* = .47). Ki^cer^, influx rate constant.

Median antipsychotic treatment received was 0.38 chlorpromazine dose years (interquartile range of 0.41). There was no significant change in whole striatal dopamine synthesis capacity (Ki^cer^) in people who received adequate treatment (baseline mean = 13.07 × 10^−3^/min, SD = 1.01 × 10^−3^/min; follow-up mean = 12.85 × 10^−3^/min, SD = 1.09 × 10^−3^/min), *t*_16_ = 0.74, *p* = .47 (Figure 1).

Data points for all subjects are given as a scatterplot in Supplemental Figure S1, categorized as responders, nonresponders (using CGI-I), and subjects taking subtherapeutic medication and those nonconcordant with antipsychotic medication.

Because aripiprazole is a partial agonist, in contrast to a full antagonist, and thus may have different effects, we repeated the analyses after excluding the 2 subjects who received aripiprazole monotherapy and repeated the analysis after excluding all subjects taking aripiprazole, including those taking it as an adjunct to a dopamine antagonist. There was no significant effect of antipsychotic treatment on Ki^cer^ after restricting the analyses to subjects taking full antagonists in either analysis (*p* = .40 and *p* = .91, respectively).

There was no significant effect of treatment on Ki^cer^ for the other regions analyzed (striatal functional subdivisions, caudate, putamen, thalamus, and substantia nigra) (see Table 3).

**Table 3:** Mean Ki^cer^ in Striatal Subdivisions and Extra-striatal Regions Before and After Antipsychotic Treatment

Brain Region	Ki^cer^ Baseline	Ki^cer^ Follow-up	*p* Value
Whole Striatum	13.07 × 10^−3^ (1.01 × 10^−3^)	12.94 × 10^−3^ (0.79 × 10^−3^)	.47
Associative Striatum	13.04 × 10^−3^ (1.07 × 10^−3^)	12.87 × 10^−3^ (0.86 × 10^−3^)	.59
Limbic Striatum	12.89 × 10^−3^ (0.90 × 10^−3^)	12.79 × 10^−3^ (0.93 × 10^−3^)	.71
Sensorimotor Striatum	13.21 × 10^−3^ (1.09 × 10^−3^)	12.83 × 10^−3^ (1.19 × 10^−3^)	.27
Caudate	11.12 × 10^−3^ (0.93 × 10^−3^)	11.93 × 10^−3^ (1.10 × 10^−3^)	.51
Putamen	14.17 × 10^−3^ (1.09 × 10^−3^)	13.91 × 10^−3^ (1.17 × 10^−3^)	.44
Thalamus	2.83 × 10^−3^ (0.32 × 10^−3^)	2.78 × 10^−3^ (0.31 × 10^−3^)	.65
Substantia Nigra	7.21 × 10^−3^ (0.81 × 10^−3^)	7.06 × 10^−3^ (1.12 × 10^−3^)	.55

Values are presented as mean (SD).

Ki^cer^, influx rate constant.

There was no change in standardized uptake value in the reference region (cerebellum) with treatment (baseline mean = 1.3 × 10^−3^/min, SD = 0.35 × 10^−3^/min; follow-up mean = 1.45 × 10^−3^/min, SD = 0.58 × 10^−3^/min), *t*_16_ = −1.27, *p* = .15. There was no change in striatal volume before and after antipsychotic treatment (baseline mean = 2006.53 mm, SD = 180.54) and follow-up (mean = 2047.94 mm, SD = 216.43), *t*_16_ = −1.39, *p* = .18. There was no effect of weight on Ki^cer^ (*r* = .08, *p* = .73).

### Relationship Between Change in Dopamine Synthesis Capacity and Symptom Change

**Figure 2:**
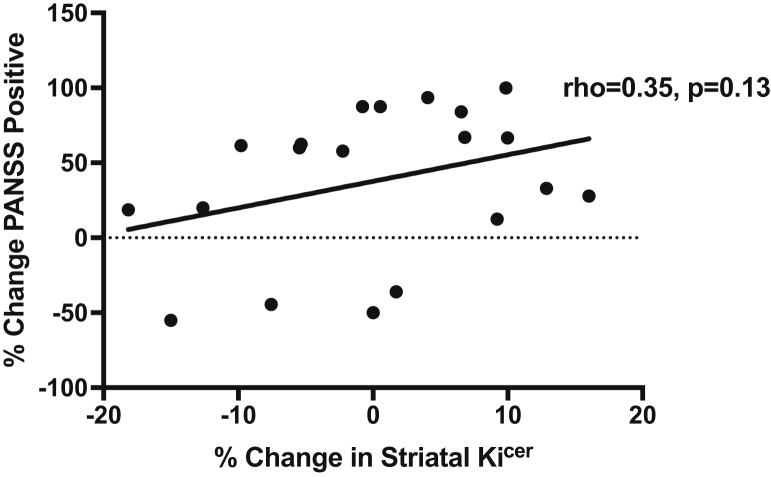
Relationship between change in dopamine synthesis capacity and percentage improvement in Positive and Negative Syndrome Scale (PANSS) positive symptoms. There is no significant correlation between improvement in PANSS positive symptom improvement and change in influx rate constant (Ki^cer^) (ρ = .35, *p* = .13).

There was no significant correlation between percentage decrease in whole striatal Ki^cer^ and percentage improvement in PANSS positive symptoms (*n* = 20, ρ = .35, *p* = .13), total symptoms (ρ = .25, *p* = .29), or negative symptoms (ρ = .10, *p* = .68) (see Figure 2).

There was no relationship between change in thalamic Ki^cer^ and change in PANSS negative symptom severity score in people taking antipsychotic treatment (*n* = 17, ρ = −.26, *p* = .31).

## Discussion

Our main findings were that there was no significant change in striatal dopamine synthesis capacity with antipsychotic treatment and no significant association between change in dopamine synthesis capacity and change in psychotic symptoms. To the best of our knowledge, this is the first study to measure change in dopamine synthesis capacity after treatment with second-generation antipsychotics.

### Effects of Antipsychotic Medication on Presynaptic Dopamine Function

Our finding of no change in presynaptic striatal dopamine function with prolonged antipsychotic treatment is consistent with acute studies in healthy volunteers, which found no significant overall change in dopamine synthesis capacity after treatment with a number of different antipsychotic drugs 53, 54, 55. However, our findings contrast with those in the only other study examining effects in a markedly smaller sample of people with schizophrenia (25), which found a decrease in dopamine synthesis capacity in caudate and putamen after a mean of 5 weeks haloperidol treatment. A possible explanation could be the relatively high dose of haloperidol (nearly double the chlorpromazine-equivalent dose in our study). Thus, taken together with our findings, this suggests that while higher doses of antipsychotics may have effects, antipsychotic treatment at doses in the range typically used in current practice is not associated with significant changes in dopamine synthesis capacity.

Our study differed from the Gründer *et al.* (25) study in terms of experimental design and methodology for quantification of F-DOPA kinetics and therefore is not directly comparable. From a methodological perspective, the two parameters of interest (k3 in the Gründer *et al.* study and Ki^cer^ in our study) are different. Ki^cer^ is related to k3 and Ki^cer^ in our study (56) but also is dependent on perfusion and tissue-to-blood tracer diffusion. One major methodological difference is the length of scan (120 minutes in the Gründer *et al.* study vs. 95 minutes in our study). There is evidence from monkey and human studies that metabolism of radiolabeled dopamine becomes appreciable by 120 minutes 57, 58. Because dopamine metabolism is not accounted for in the analysis used by Gründer *et al.* (25), this could introduce noise into the measurement of k3.

It is also worth noting that Gründer *et al.* (25) found, after haloperidol, an increase in absolute terms of radiolabeled DOPA volume of distribution and reduction in radiolabeled DOPA from striatal tissue back across the blood-brain barrier. We did not measure these parameters, although we would expect alterations in them to alter Ki^cer^ if antipsychotic treatment had altered them in our study, so this is unlikely to explain our findings.

Microdialysis findings show that antipsychotic treatment acutely induces a transient increase in striatal extracellular dopamine levels, which fall back to basal levels with chronic treatment (24). Our results are consistent with these findings and extend them to show that, in addition to having no lasting effect on extracellular dopamine levels, chronic treatment does not alter dopamine synthesis capacity. It should be recognized that we did not measure dopamine neuron firing, and so our findings cannot exclude an effect on midbrain dopamine neuron firing, as seen in the electrophysiology studies of chronic antipsychotic treatment (20). Determining this will require preclinical studies to test the relationship among dopamine neuron firing, striatal dopamine levels, and dopamine synthesis capacity.

We did not find a change in striatal volume with antipsychotics in our study. A systematic review of the effects of second-generation antipsychotics, such as those used in our study, found inconsistent effects on striatal volume, with some studies reporting increases, others finding decreases, and others finding no effects, as was the case in our study (59).

### Limitations

Our study was powered to detect a statistically significant difference and relationship between change in symptoms and change in dopamine synthesis capacity of the size reported by Gründer *et al.* (25). While a type II error remains possible, the data suggest that any effect is unlikely to be clinically significant. It should be acknowledged that the follow-up at 6 months showed that the patient group included patients who subsequently met diagnostic criteria for bipolar affective disorder as well as schizophrenia. It is often not possible to disentangle diagnoses at baseline, although we should emphasize that criteria for study entry included the presence of psychosis, according to criteria used before to define psychosis in first-episode illness (60) and requiring antipsychotic treatment (61).

Because our study was naturalistic in design, we were unable to test the effects of one specific antipsychotic and accept that some antipsychotics used in this study (e.g., amisulpride) have more selectivity for D_2/3_ receptors. Nonetheless, by focusing on antipsychotic response and including only people taking antipsychotics at a dose that would block D_2/3_ receptors, we examined a potential common mechanism by which antipsychotics might act.

It should be noted that 4 hours after smoking a cigarette could correspond to the period of subjective nicotine withdrawal, although nicotine’s occupancy of nicotinic receptors in the brain remains high (40).

It is conceivable that antipsychotics could have had an effect on blood flow (62) and effects on tracer delivery to the reference region (cerebellum). Our finding of no difference in standardized uptake value in the cerebellum suggests that this is not a significant issue, although further studies are required to exclude this and investigate local blood flow changes. Moreover, it is unlikely to account for our findings given that this would require opposite blood flow effects in the cerebellum to the striatum to account for no overall change. Ideally, tests of the biological effect of antipsychotic drugs would involve randomizing patients to placebo treatment as well as excluding nonspecific effects of treatment. This would have ethical implications and therefore is unlikely to be feasible.

### Implications for Clinical Care

Our main finding, that antipsychotics do not alter dopamine synthesis capacity in people presenting with first-episode psychosis, extends cross-sectional evidence that dopamine synthesis capacity remains elevated, even in patients on long-term antipsychotic treatment 12, 63, to indicate that antipsychotics do not normalize the major (presynaptic) dopaminergic abnormality seen in the disorder. This provides a potential neurobiological explanation for why psychosis recurs after antipsychotic treatment is stopped (64) and, potentially for continued treatment, suggests that stopping dopamine receptor blockade would lead to dopamine dysfunction’s being unmasked.

In conclusion, antipsychotic medication does not alter dopamine synthesis capacity in people with first-episode psychosis, and change in symptoms is not associated with change in dopamine synthesis capacity. This indicates that therapeutic effects of antipsychotic medication are not due to altering dopamine synthesis capacity.
