# Supplementary material for: The Effects of Antipsychotic Treatment on Presynaptic Dopamine Synthesis Capacity in First-Episode Psychosis: A Positron Emission Tomography Study
Source: Biol Psychiatry. 2018 Jul 11;85(1):79–87. doi: 10.1016/j.biopsych.2018.07.003 (PMC6269123; doi:10.1016/j.biopsych.2018.07.003)
Supplement: Supplemental Table S1 and Supplemental Figure S1 [file mmc1.pdf]

The Effects of Antipsychotic Treatment on Presynaptic Dopamine Synthesis Capacity in First Episode Psychosis: A Positron Emission Tomography Study

Supplemental Information

Supplementary Table S1. Psychotropic medication received

Patients who were adherent to antipsychotic treatment, N=17<br>Medication
Amisulpride, N=6
Amisulpride and Aripiprazole, N=1
Amisulpride then Quetiapine, N=1
Aripiprazole, N=2
Aripiprazole then Quetiapine, N=1
Olanzapine and Aripiprazole, N=1
Quetiapine, N=2
Lurasidone, N=1
Risperidone, N=1
Risperidone and Aripiprazole, N=1

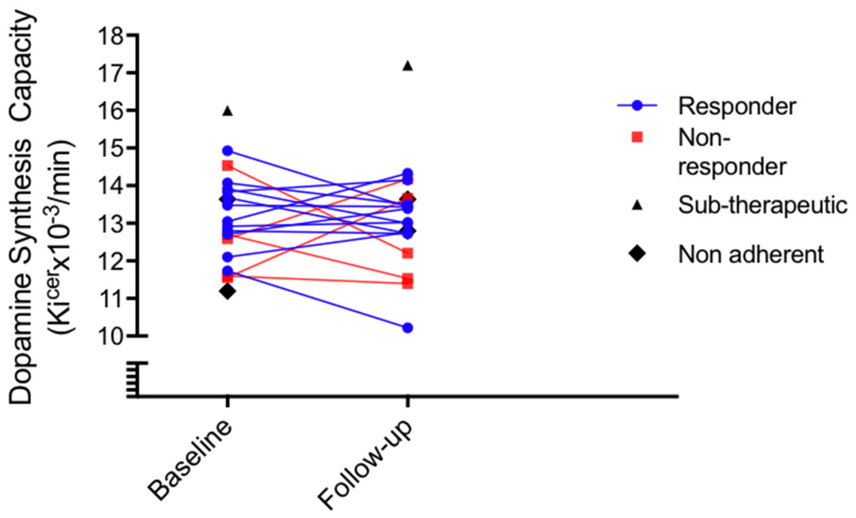

Supplemental Figure S1. Scatterplot of whole striatal Ki^{cer} values at baseline and at follow-up.
